# Molecular characteristics of mismatch repair genes in sporadic colorectal tumors in Czech patients

**DOI:** 10.1186/1471-2350-15-17

**Published:** 2014-01-31

**Authors:** Veronika Polakova Vymetalkova, Jana Slyskova, Vlasta Korenkova, Ludovit Bielik, Lucie Langerova, Pavel Prochazka, Alexandra Rejhova, Lucie Schwarzova, Barbara Pardini, Alessio Naccarati, Pavel Vodicka

**Affiliations:** 1 Institute of Experimental Medicine Academy of Sciences of the Czech Republic, Prague, Czech Republic; 2 Institute of Biology and Medical Genetics, First Faculty of Medicine Charles University, Prague, Czech Republic; 3 Institute of Biotechnology, Academy of Sciences of the Czech Republic, Prague, Czech Republic; 4 Faculty of Science, Charles University, Albertov 6, Prague, Czech Republic; 5 Human Genetics Foundation, Turin, Italy

**Keywords:** Colorectal cancer, Mismatch repair genes, Expression levels, Promoter methylation

## Abstract

**Background:**

Mismatch repair (MMR) genes are known to be frequently altered in colorectal cancer (CRC). Both genetics and epigenetics modifications seems to be relevant in this phenomenon, however it is still not clear how these two aspects are interconnected. The present study aimed at characterizing of epigenetic and gene expression profiles of MMR genes in sporadic CRC patients from the Czech Republic, a country with one of the highest incidences of this cancer all over Europe.

**Methods:**

Expression levels and CpG promoter methylation status of all MMR genes were evaluated in DNA from tumor and adjacent mucosal samples of 53 incident CRC patients*.*

**Results:**

We have found significantly increased transcription levels in *EXO1* gene in tumor tissues (P = 0.05) and significant over-expression of *MSH3* gene in colon tumors when compared to adjacent mucosal tissues (P = 0.02). Interestingly, almost all MMR genes were differently expressed when localization of tumors was compared. In particular, colon tumors showed an up-regulation of *EXO1, MSH2, MSH3, MSH6,* and *PMS2* genes in comparison to rectal tumors (P = 0.02). Expression levels of all MMR genes positively correlated between each other. The promoter methylation of *MLH1* gene was observed in 9% of CRC tissues only*.*

**Conclusions:**

In our study, we have observed different pattern of MMR genes expression according to tumor localization. However, a lack of association between methylation in MMR genes and their corresponding expressions was noticed in this study, the relationship between these two aspects is worthy to be analyzed in larger population studies and in pre-malignant stages.

## Background

Colorectal cancer (CRC) represents a serious health problem in the Central Europe and particularly in the Czech Republic, where the incidence for colon and rectal cancers ranks the third and the first highest worldwide, respectively [1,2]. Notably, rates among males in the Czech Republic and Japan have already exceeded the peak of incidence observed in the United States, Canada, and Australia, where rates are declining or stabilizing [3,4]. The reasons for such frequencies are unknown. It is generally accepted that the etiology of CRC is multifactorial, involving hereditary and environmental factors, as well as somatic changes occurring during tumor progression [5].

There are at least three distinct, and relatively discrete, molecular pathways associated with this disease: chromosomal instability (CIN), microsatellite instability (MSI) and the cytosine phosphate guanine (CpG) island methylator phenotype (CIMP). The development of MSI pathway (15% of CRC) is considered to be due to defective DNA mismatch repair (MMR) system [6]. In CIMP, a number of genes become transcriptionally silenced due to hypermethylation of their promoters, and this represents a key epigenetic mechanism of inactivation of tumor suppressor genes [7], as well as MMR genes. Impaired MMR system, arising frequently from aberrant methylation of gene promoters, may be detectable by an altered gene and protein expression patterns. Aberrant DNA methylation - hypermethylation and hypomethylation compared to normal tissue - has been associated with a large number of human malignancies, including CRC [8]. DNA hypomethylation, prevalent as a genome-wide event, usually occurs in more advanced stages of tumor development whereas DNA hypermethylation is often observed as a discrete, targeted event within tumor cells, resulting in specific loss of gene expression [9].

Based on a number of relatively large case–control and prospective cohort studies, ∼30–40% of sporadic proximal-site colon cancers are CIMP positive, compared to 3–12% of distal colon and rectal cancers [10,11]. Thus, CIMP is attributable to tumors of the proximal colon, independently of MSI status. CIMP has been also associated with *BRAF* mutations in both microsatellite stable and unstable colon cancers [11,12]. It has been suggested that there are two general types of CIMP in sporadic tumors: CIMP high, related to *BRAF* mutations and *MLH1* methylation, and CIMP low, related to *KRAS* mutations [13].

Approximately 90% of all MMR alterations/modifications in sporadic CRC cases are most commonly caused by epigenetic inactivation of *MLH1* or *MSH2* gene [14]. The hypermethylation of the *MLH1* promoter is also the predominant cause of MSI high (MSI-H) in sporadic tumors [15]. MSI-H cancers with methylated *MLH1* are distinct from the rest of CRC by delayed onset and association with the female gender [16]. On the other hand, CRC cases without altered MMR genes show low-frequency MSI (MSI-L) or are microsatellite stable (MSS) [14]. Moreover, gene alterations of other MMR genes may also be involved in the CRC progression [17]. Goel et al. [18] assumed that germline hypermethylation of *MLH1* and *MSH2* may serve as predisposing events in some CRC cases.

The Czech Republic has one of the highest reported incidences of CRC worldwide, thus the analysis of mRNA expression profile of MMR genes and their epigenetic characterization has a great meaning. Apparently, the clarification of a potentially abnormal epigenetic profile of tumor tissues, as well as their genetic constitution, could contribute to better classify the CRC cases or could ultimately result in the improvement of therapy.

Thus the aim of our study was to investigate the epigenetic characteristics and gene expression profiling of MMR genes in tumors of CRC patients of Czech nationality, with respect to their clinical and histopathological characteristics. We have hypothesized that the high incidence of CRC in this country could be due to different genetic and epigenetic pattern of DNA repair genes, which could reflect possible specific geographical, ethnic, dietary or lifestyle factors.

## Methods

### Patients’ characteristics and collection of biological specimen

Fifty three patients with sporadic CRC were recruited between 2009 and 2011 at the Thomayer Hospital and at the General University Hospital (both located in Prague, Czech Republic), where they underwent surgical resection. All patients signed an informed consent. Ethics approval was granted by the committees of the above hospitals.

From each patient, tumor tissue and adjacent mucosal colon/rectal tissue (5–10 cm distant from the tumor) were resected and deep frozen immediately after removal. Peripheral blood was taken a day before surgery and stored at 4°C until processing that was no longer than 3 hours. The clinical stage of patients at diagnosis was classified according to the tumor–node–metastasis (TNM) system according to UICC (Union for International Cancer Control).

Tumor and adjacent mucosal tissues were homogenized by MagNA Lyser (Hoffmann-La Roche). Genomic DNA and mRNA were isolated from tumor tissues and adjacent mucosal tissue with AllPrep DNA/RNA Isolation Kit protocol according to the manufacturer’s instructions (Qiagen, Hilden, Germany).

### MSI status

MSI was assessed by multiplex PCR with pseudomonomorphic mononucleotide markers BAT-25, BAT-26, NR-21, NR-24, NR-27 using primers labeled with FAM, HEX, or NED followed by analysis of PCR products on 5% denaturing gel electrophoresis on ABI PRISM 310 System (Applied Biosystems) as described previously by [19].

Tumor DNA samples were compared, and tumors showing instability at one or two locus were scored as MSI-low (MSI-L), at three or more loci as MSI-high (MSI-H). Genescan software was used for calculation of the size of each fluorescent PCR product.

### Gene expression profiling

#### Gene selection

A panel of all MMR genes was extracted from the complete list of all DNA repair genes organized by pathways available online (http://sciencepark.mdanderson.org/labs/wood/DNA_Repair_Genes.html#MMR). Eleven genes (*EXO1*, *MLH1, MLH3, MSH2, MSH3, MSH4, MSH5, MSH6, PMS1, PMS2,* and *PMS2L3*) were analyzed for mRNA expression levels.

#### Sample preparation

The total RNA was measured on ASP-3700 UV/Vis Spectrophotometer (Avans-Biotechnology, Taiwan) for quantity control and OD_260/280_ ratio for an indication of nucleic acid purity. RNA integrity number (RIN) was checked using Agilent Bioanalyzer 2100, with RNA 6000 Nano Assay (Agilent Technologies). Each pair of tumor/adjacent mucosal tissue did not differ by more than ±2 RIN units. The complementary DNA (cDNA) was obtained from 1 μg of total RNA by using First strand cDNA synthesis kit (MBI Fermentas, Vilnius, Lithuania). All samples were tested to exclude possible inhibition of the quantitative PCR (qPCR) reaction by spiking with RNA from an extraction control kit (TATAA, Sweden). cDNA was diluted to 10 ng/mL and preamplified for 18 cycles on a Bio-Rad CFX96 Real Time PCR Instrument (Bio-Rad) with TaqMan Preamp Master Mix (Applied Biosystems) according to the manufacturer’s protocol. qPCR was conducted using the high-throughput platform BioMark HD System (Fluidigm). Five μL of Fluidigm sample premix consisted of 1 μL of 20× diluted preamplified cDNA, 0.25 μL of 20×Gene Expression (GE) sample loading reagent (Fluidigm), 2.5 μL of TaqMan universal mastermix II without uracil-N glycoslyase (UNG; Life Technologies), and 1.25 μL of RNase/DNase-free water. Each sample premix was combined with 5 μL FAM-MGB assays (Primer Design) at a final concentration of 300 nmol/L and 2.5 μL 2x Assay loading reagent (Fluidigm). The reaction volume for a single qPCR reaction was 6.7 nL. Thermal conditions for qPCR were: 95°C for 10 minutes, 45 cycles of 95°C for 15 seconds, and 60°C for 60 seconds. Actin beta (ACTB) and 18S rRNA were used as reference genes selected from a geNorm reference genes selection kit (Primer Design) by Normfinder (GenEx Enterprise).

#### qPCR data pre-processing

Data were collected from 2 GE Dynamic Arrays 96.96 (Fluidigm) and pre-processed in GenEx Enterprise software (MultiD). Interplate calibration was conducted and the technical replicates were averaged. Cut-off value for Cq was set at 25. The Cq 25 measured in BioMark system would approximately correspond to Cq 35 at the conventional qPCR cyclers [20]. When more than 12% of the data were missing for each sample/gene due to a very low expression and low fluorescence signal, the particular sample/gene was removed from the dataset. As a result of this selection *MSH4, MSH5* and *PMS2L3* genes were excluded from analyses. Data were normalized to reference genes, recalculated to relative quantities with the lowest expression set to 1, and transformed to log2 scale.

### Promoter CpG islands methylation profiling

#### Methylation-specific PCR

A prediction of CpG islands site within the promoter region of MMR genes was carried out by screening with CpG Islands Searcher (http://cpgislands.usc.edu/). Genomic DNA was treated with sodium bisulfite using the Epitect Whole Bisulfitome Kit (Qiagen, Hilden, Germany). Methylation-specific PCR (MSP) analysis of bisulfite-converted DNA was conducted using the Epitect MSP kit (Qiagen, Hilden, Germany), following the producer’s protocols. Primers (Sigma-Aldrich; Additional file 1: Table S1a) specific for methylated and unmethylated bisulfite-converted DNA were designed for investigated genes by applying MethPrimer algorithm [21]. To test whether promoter methylation can affect the mRNA expression levels of MMR genes, only those genes which were successfully analyzed for the gene expression were subsequently considered. MSP reactions were performed as previously described by [22].

#### Methylation-sensitive high resolution melting

Methylation-sensitive high resolution melting (MS-HRM) was conducted only on those samples that showed positive results in MSP, to validate the observations. Whole genomic DNA was treated with sodium bisulfite using the Epitect Bisulfite Kit (Qiagen, Hilden, Germany) to convert unmethylated cytosines to uracils, following the manufacturer’s protocol, as described in [23]. Real-time PCR followed by HRM was carried out in high-performance Eco Real-Time PCR system (Illumina, San Diego, CA, USA). Primer sequences for *MLH1* were described earlier [23,24], while for *MLH3* primers (Additional file 1: Table S1b) were designed using Methyl Primer Express Software v1.0 (Applied Biosystems, Foster City, CA, USA). The reaction mixture (10 μl final volume) consisted of 10 ng of template DNA, 1× EpiTect HRM Master Mix (Qiagen) and 300 nmol/l of each primers. PCR was initiated by incubation at 95°C for 5 min, followed by 50 cycles at 95°C for 10 sec, 56°C for 20 sec, 72°C for 10 sec. For each assay, a standard dilution series of EpiTect Control DNAs (Qiagen) was run to assess the quantitative properties and sensitivity of the assay. Fluorescence data were converted into melting peaks by the Eco Software (Illumina, Ver. 3.0.16.0). The cut-off value for aberrant methylation was set to 25% or higher.

### Statistical analyses

Statistical analyses were conducted by IBM SPSS Statistics 18, GenEx Enterprise and SAS 9.2 software. Expression levels of all studied genes did not follow a normal distribution in the study population, as analyzed by Kolmogorov–Smirnov test. Data were logarithmically transformed and nonparametric tests were used for statistical analyses; for comparison of medians, Mann–Whitney test was applied. Correlations were determined by a Spearmen correlation coefficient. All statistical tests were conducted at a 95% confidence level.

## Results

### Population characteristics

The detailed patient’s characteristics are shown in Table 1. The study group included 38 men and 15 women with a mean age of 67.0 (±10.6) years. Twenty six patients had a tumor localized in the colon and 27 in the rectum. Seven patients were diagnosed with pathologic stage I, 27 with stage II, 11 with stage III and 8 with stage IV. All tumors were histologically confirmed as adenocarcinomas. Three patients had tumor of a well-differentiated grade, 43 a moderately differentiated and 7 patients poorly differentiated.

**Table 1:** Patients’ characteristics

	**N (%)**
**Gender**	
Male	38 (71.7)
Female	15 (28.3)
**Age at diagnosis (years)**	
Mean ± SD	67.0 ±10.6
**Diagnosis**	
Colon cancer	26 (49.1)
Rectal cancer	27 (50.9)
**pTNM status**	
I	7 (13.2)
II	27 (50.9)
III	11 (20.8)
IV	8 (15.1)
**Grade**	
Well-differentiated	3 (5.7)
Moderately differentiated	43 (81.1)
Poorly differentiated	7 (13.2)
**MSI status**	
MSS	47 (88.7)
MSI-H	6 (11.3)
**MSI status in colon cancer**	
Proximal colon	5 (83.3)
Distal colon	1 (16.7)

Ten patients with rectal cancer received neoadjuvant therapy before surgery.

### MSI status

Tumor tissue of 6 patients displayed MSI. For all of them, 3 and more loci showed nucleotide expansions and they were therefore considered to be MSI-H. All six patients were diagnosed for colon cancer. No association of MSI status with gender, age, TNM and grade was observed.

### Expression of MMR genes

**Figure 1:**
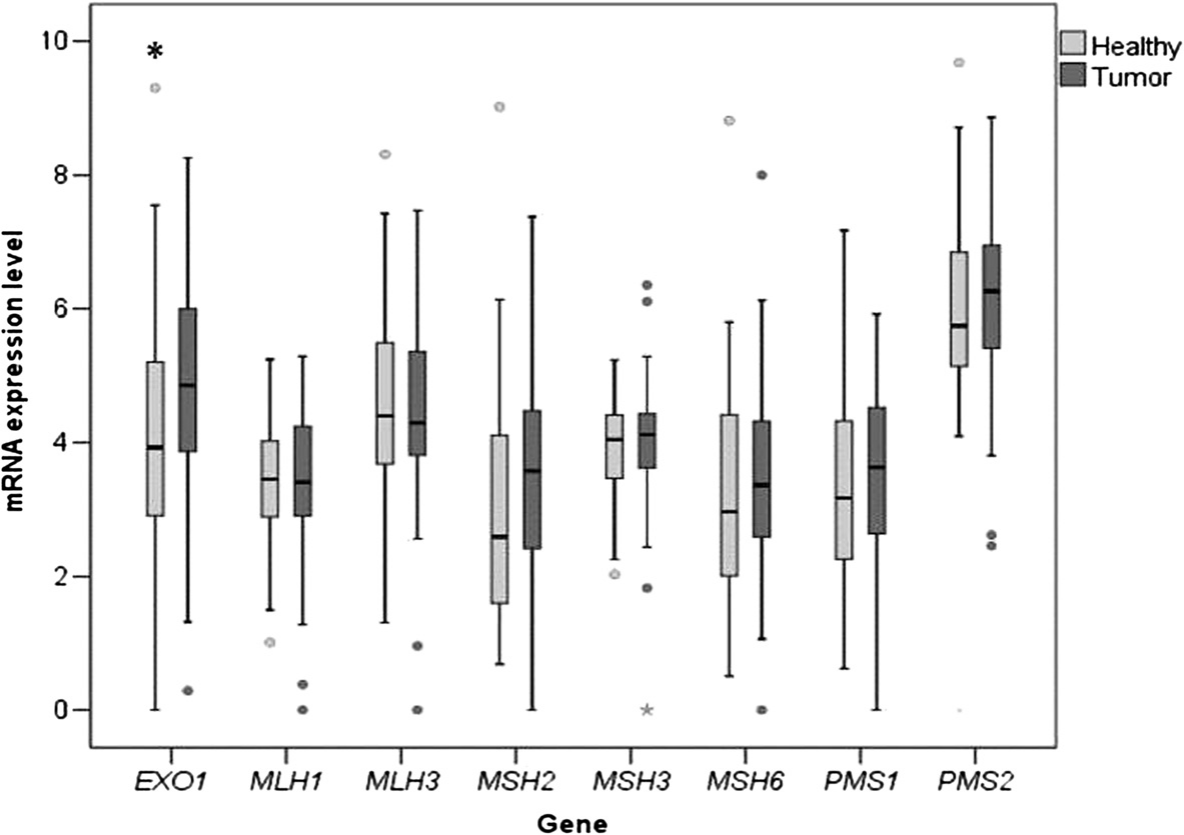
mRNA expression levels of MMR genes in tumor and adjacent mucosal tissues in the study subjects, expressed as quantities relative to the lowest detected expression assigned as value 1.

Expression levels of 8 MMR genes were successfully analyzed (Figure 1, Additional file 1: Table S2). Three other MMR genes (*MSH4, MSH5* and *PMS2L3)* were excluded from final analyses due to a very low expression and low fluorescence signal.

Overall, only *EXO1* was differentially expressed in our study group: significantly higher mRNA levels were observed in tumor tissues when compared with adjacent mucosal tissue (1.16-fold; P = 0.048; Figure 1).

After stratifying patients according to the tumor localization, a different pattern for colon and for rectal carcinomas was observed. Specifically, significantly higher expression levels of *MSH3* gene were observed in colon tumors when compared to adjacent mucosal tissue (1.18 fold change, P = 0.02; Additional file 1: Table S3b). No differences in gene expression were observed in rectal tumors when compared to adjacent mucosal tissues.

**Figure 2:**
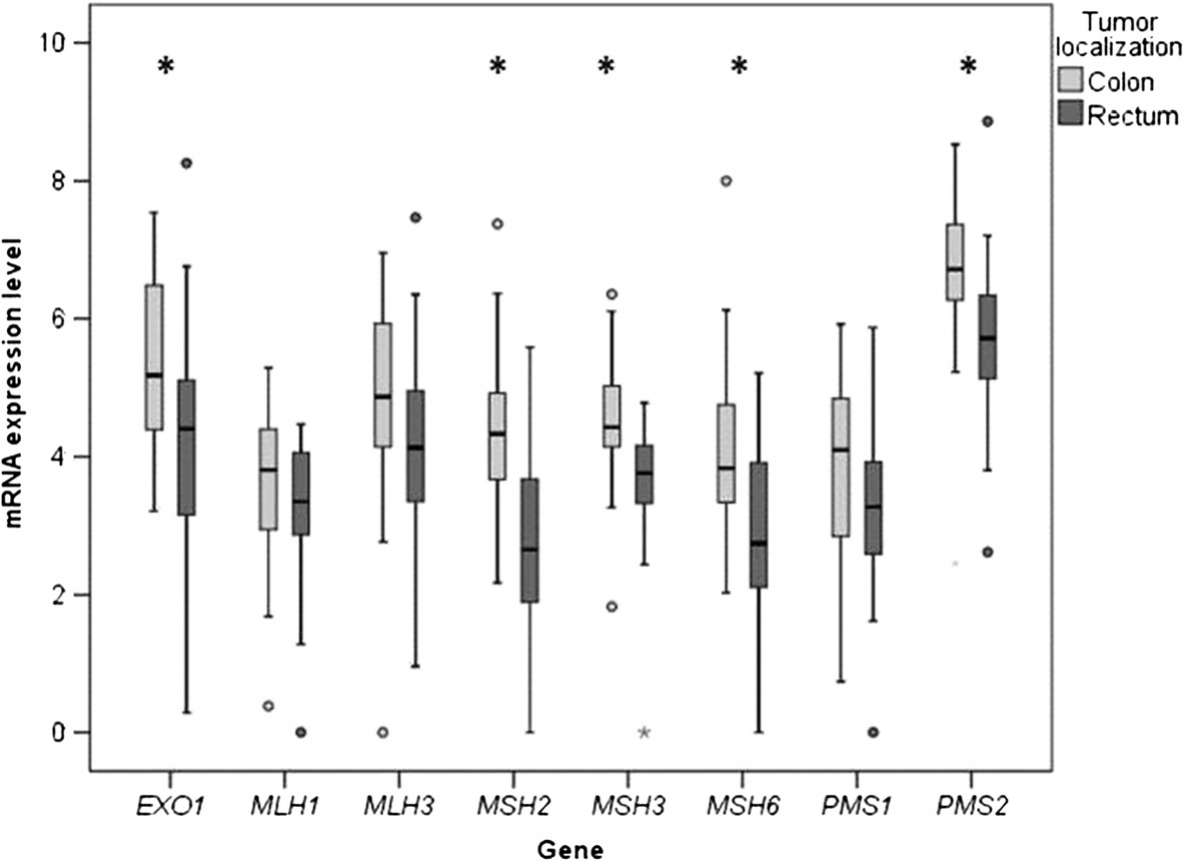
mRNA expression levels of MMR genes in colon and rectum tumors, expressed as quantities relative to the lowest detected expression assigned as value 1.

Interestingly, when expression levels of only tumor tissues with different localization were compared, tumor localized in colon showed significantly increased levels of almost all analyzed MMR genes, namely *MSH2* (1.68, P < 0.0001)*, MSH3* (1.27, P = 0.001)*, MSH6* (1.43, P = 0.004)*, PMS2* (1.16, P = 0.005) and *EXO1* (1.30 fold change, P = 0.02) (Figure 2; Additional file 1: Table S3a). Although less robust, the same tendency was also observed in the adjacent mucosal tissue for *MSH2* (1.65 fold change, P = 0.02) and *PMS2* (1.18, P = 0.03) genes. Further stratification for left and right colon did not show any differences (data not shown).

The expression pattern of investigated MMR genes was irrespective of other clinical features (pTNM staging and tumor differentiation). The only exception was for *PMS2* gene in colon tumors, where patients with the pTNM stage I + II had significantly lower expression levels than those with pTNM III + IV (−1.13, P = 0.01; data not shown). No difference was observed when patients were stratified for any of the considered demographic factors (e.g. age, sex).

Ten patients with rectal cancer (18.9%) received neoadjuvant therapy before surgery. However, none of the analyzed genes differed in the expression levels among rectal cancer patients irrespectively of the neoadjuvant therapy.

### Relationships of expression levels among different MMR genes

MMR genes positively correlated between each other in both tumor and adjacent mucosal tissues (Tables 2 and 3). The most pronounced significant correlations in tumors were observed between *MLH3* and *PMS1* or *PMS2* (R = 0.822 and R = 0.903, P < 0.0001 for both); between *MSH2* and *MSH6* (R = 0.854, P < 0.0001), and both *PMS1* and *PMS2* (R = 0.835, P < 0.0001) (Table 2).

**Table 2:** Correlations between mismatch repair genes in tumor tissues

**Gene**	**Exo1**	**Mlh1**	**Mlh3**	**Msh2**	**Msh3**	**Msh6**	**Pms1**	**Pms2**
Exo1	1							
Mlh1	0.244 (0.115)	1						
Mlh3	0.721 (***0.000***)	0.395 (**0.009**)	1					
Msh2	0.577 (***0.000***)	0.285 (0.064)	0.601 (***0.000***)	1				
Msh3	0.125 (0.423)	0.495 (***0.001***)	0.268 (0.082)	0.478 (***0.001***)	1			
Msh6	0.648 (***0.000***)	0.177 (0.257)	0.695 (***0.000***)	0.854 (***0.000***)	0.247 (0.110)	1		
Pms1	0.711 (***0.000***)	0.368 (**0.015**)	0.822 (***0.000***)	0.593 (***0.000***)	0.191 (0.220)	0.670 (***0.000***)	1	
Pms2	0.783 (***0.000***)	0.381 (**0.012**)	0.903 (***0.000***)	0.745 (***0.000***)	0.342 (**0.025**)	0.784 (***0.000***)	0.835 (***0.000***)	1

R and P value (in brackets), Significant differences are in bold, differences significant after Dunn–Bonferroni correction (P < 0.0032) are in italics.

**Table 3:** Correlations between mismatch repair genes in adjacent mucosal tissues

**Gene**	**Exo1**	**Mlh1**	**Mlh3**	**Msh2**	**Msh3**	**Msh6**	**Pms1**	**Pms2**
Exo1	1							
Mlh1	0.323 (**0.035**)	1						
Mlh3	0.849 (***0.000***)	0.362 (**0.017**)	1					
Msh2	0.758 (***0.000***)	0.230 (0.139)	0.715 (***0.000***)	1				
Msh3	0.046 (0.771)	0.511 (***0.000***)	0.166 (0.289)	0.281 (0.068)	1			
Msh6	0.849 (***0.000***)	0.230 (0.192)	0.857 (***0.000***)	0.880 (***0.000***)	0.054 (0.731)	1		
Pms1	0.814 (***0.000***)	0.399 (**0.008**)	0.887 (***0.000***)	0.630 (***0.000***)	0.100 (0.525)	0.769 (***0.000***)	1	
Pms2	0.793 (***0.000***)	0.367 (**0.016**)	0.931 (***0.000***)	0.658 (***0.000***)	0.135 (0.390)	0.782 (***0.000***)	0.855 (***0.000***)	1

R and P value (in brackets), Significant differences are in bold, differences significant after Dunn–Bonferroni correction (P < 0.0032) are in italics.

Similarly to cancer tissues, in adjacent mucosal tissues, *MLH3* correlated with *PMS1* and *PMS2* (R = 0.887 and R = 0.931, P < 0.000, respectively), but, additionally, *MLH3* also correlated with *MSH6* (R = 0.857, P < 0.0001). In addition, *MSH2* again correlated with *MSH6* (R = 0.880, P < 0.0001) and *PMS1* and *PMS2* (R = 0.855, P < 0.0001). Concerning the *EXO1,* in adjacent mucosal tissue the gene strongly correlated with *MLH3, MSH6* and *PMS1* (R = 0.849, R = 0.849, and R = 0.814, P < 0.0001 for all) (Table 3).

### Promoter methylation of MMR genes

The promoter methylation status was analyzed only in those genes whose expression analyses were successfully conducted. Therefore, methylation levels of promoter regions of *EXO1*, *MLH1, MLH3, PMS1, PMS2, MSH2, MSH3,* and *MSH6* genes were evaluated in DNA from tumor and adjacent mucosal tissues of 53 CRC patients*.* Methylation of promoter region was detected only in *MLH1* (Additional file 2: Figure S1). For this gene, a fragment of 100 bp length containing 8 CpG sites was analyzed. Five tumors and two samples from adjacent mucosal tissues exhibited methylated *MLH1* promoter region. In patients, where normal tissues presented *MLH1* promoter methylation, tumor tissue also exhibited promoter methylation.

The investigated promoter region resulted methylated in MSI-H colon tumors only (p < 0.00001)*.*

We did not observe any statistical significant variation in mRNA expression levels of *MLH1* gene in the patients with promoter methylation when compared to patients without promoter methylation.

## Discussion

Genetic and epigenetic alterations underlie the pathogenesis of cancer. In particular, the disruption of epigenetic mechanisms leads to abnormal development of cells and is involved in malignant transformation [25]. Variations in DNA methylation are important epigenetic modifications which may affect gene expression by modifying the DNA structure without altering the native nucleotide sequence.

In the present study, we have analyzed the mRNA expression of MMR genes and their promoter methylation in CRC tissues in patients from the Czech Republic, a country that has the second highest CRC incidence and mortality among 38 European countries [1]. With the exception of *MLH1* and *MSH2* genes, the methylation status of the other MMR genes was globally less studied or even never previously analyzed.

In our study, we observed the promoter methylation of *MLH1* gene only. Notably, this modification was observed in MSI-H colon tumors only, as it is generally observed in CRC [14]. The incidence of *MLH1* promoter methylation observed in our study is in concordance with another Czech study of Vasovcak et al. [26], where mutational profiles of CRC high risk genes together with methylation of *MLH1* gene were analyzed. Similarly to the work of Vasovcak et al. [26], any *MLH1* promoter methylation was not detected in rectal tumors. Rare *MLH1* promoter methylation in rectal cancers was described also in the study of Samowitz et al. [27], but it was accompanied by high degree of MMR protein deficiency, possibly due to the inclusion of Lynch associated tumors. The reason for the differential *MLH1* promoter methylation and tumor localization is still unknown and can be caused by dietary habits, different environment (e.g. varying pH) in different parts of the colon, or by the combination of both aspects [26] or by other factors which could affect the presence of promoter methylation, like presence of bacterial flora [28]. In our study, the presence of promoter methylation of *MLH1* gene was also not related to the mRNA levels. This lack of association could be due to the small size of the population. An inverse correlation to *MLH1* expression was observed in a previous study of Oster et al. [24] and may have different explanation. Methylation of *MLH1* gene might be explained by the fact that only few CpG sites were interrogated, and the interrogated sites may not be the sites involved in regulation of the gene. In addition, the presence of alternative transcription start sites may also be involved. Recently Jones [29] summarized that genes silenced by Polycomb complexes are much more likely than other genes to become methylated in cancer and thus a silent state could even precede methylation. Thus, the evidence regarding the timing of DNA methylation could be consistent with the idea that methylation adds an additional level of stability to epigenetic states.

In patients where the tumor and normal tissues presented *MLH1* promoter methylation, blood samples were also analyzed to confirm potential germline hypermethylation. However, in our study, we did not observe any *MLH1* promoter methylation in the DNA from blood (data not shown). This result pointed to somatic origin of *MLH1* promoter methylation in our study.

Although rather small (1.16-fold), we also observed different expression levels for *EXO1* gene when compared tumor and adjacent mucosal tissues. Higher expression levels for *EXO1* in tumor tissues are in agreement with the study of Ioana et al. [30]. Although, data in that study were normalized to a different reference gene, *GAPDH*, and the investigated population was also smaller than ours. Recently, Caradec et al. [31] suggested not to use *GAPDH* as a reference gene for normalization in CRC experiments, since it appears to be among the most variable. Other authors showed that *GAPDH* expression varies according to oxygen tension and hypoxia, critical factors in cancer development, especially in CRC [32]. On the other hand, Ide et al. [33] observed a lower mRNA level of MMR genes in tumor samples as compared with the normal tissue. In our study, *MSH3* gene had significantly higher expression levels in colon tumors when compared to adjacent mucosa. Tentori et al. [34] observed that defective expression of the protein MSH3 is frequently detected in colon cancer. Higher expression levels were found in tumors of the colon when compared to those in the rectum. These differences were more pronounced in *EXO1, MSH2, MSH3, MSH6*, and *PMS2* genes. The same tendency was observed in adjacent mucosa for *MSH2* and *PMS2* genes. Our results may suggest different mechanisms in the genesis of colon and rectal cancers as it was already postulated by [35]. The reason for higher mRNA levels of MMR genes in colon could be actually due to the fact that stools are kept in the colon for a longer time than in the rectum. In this way, colon is more exposed to various carcinogens from the food, and thus needs more protection against the carcinogenic events. Higher expression levels of DNA repair genes could be one of these mechanisms of protection. As tumor localization was the major factor influencing gene expression, location-specific analysis may identify location-associated pathways and enhance the accuracy of class prediction.

In the present study, we have also observed a strong relationship between between *EXO1* expression and those of genes involved in the MutSα heterodimer (*MSH2-MSH6*). Previously, Jiricny [36] also noticed that decreased activity of *EXO1* is accompanied with the low concentrations of genes involving in the MutSα heterodimer.

We observed a strong correlation between expressions of *MSH2* and *MSH6* genes. Vageli et al. [37] recently demonstrated that reduction of *MSH6* mRNA levels is a frequent event in bladder tumorigenesis and reflects a common mechanism of suppression with *MSH2.* Another MMR heterodimer, MutLα, consisting of *MLH1* and *PMS2,* positively correlated, but the strength of such correlation was considerably lower than that for the MutSα heterodimer. Interestingly, the strongest correlations were observed between *MLH3* and *PMS1* and *PMS2*. This is the first time that a correlation between above genes is reported. Previous observations indicated that *PMS2* gene is required for the correction of single-base mismatches, and *PMS2* and *MLH3* contribute both to the correction of insertion-deletion loops resulting from DNA replication, DNA damage or from recombination events between non-identical sequences during meiosis [38]. The role of *PMS1* in MMR still awaits further clarification, but it is assumed that coordinates the downstream processes after mismatch recognition by MutSα heterodimer together with *MLH1*[39].

DNA repair pathways are a part of a multistep, multifactorial process to remove the damaged DNA sequence and to resynthesize particular part of the DNA strand. Thus, interplay exerted by multiple genes is crucial and more informative for identifying genes responsible for human cancer. Analyzing the difference in expression, individual variability, and co-expression in our study has provided an initial characterization of the MMR pathway and can help in further understanding of the cellular DNA repair system in human CRC.

A lack of association between methylation in MMR genes (representing rather low-frequency events) and their corresponding expressions could be due to the small size of the population. Above aspect emerges therefore as a main limitation of the present study.

## Conclusions

In summary, our combined genetic and epigenetic analysis confirmed some previous data from other studies on CRC patients, but also provided novel findings. First, a strong correlation was observed either between *MLH3* and *PMS1* or *PMS2*. Second, although we did not confirm the expected traits of deregulation of MMR genes in sporadic CRC in patients recruited in the Czech Republic, we have found interesting and strong differences in expression of almost all MMR genes when colon and rectal tumors were compared. This finding might point to the distinct genesis of both neoplasia. Outcomes of the present study will require further validation with functional assays, particularly in tumors and in larger population study.

## Abbreviations

ACTB: Actin beta; cDNA: Complementary DNA; CIMP: Cytosine phosphate guanine island methylator phenotype; CIN: Chromosomal instability; CpG: Cytosine polyguanine; CRC: Colorectal cancer; MMR: Mismatch repair; MSI: Microsatellite instability; MSI-H: MSI high; MSI-L: MSI low; qPCR: Quantitative PCR; TNM: Tumor-node-metastasis; UICC: Union for International Cancer Control; UNG: Uracil-N glycoslyase.

## Competing interests

The authors declare that they have no competing interests.

## Authors’ contributions

VPV was responsible for drafting the manuscript, methylation specific PCR and participated in the whole design of the study. SJ performed the statistical analysis and interpretation of data. KV carried out the qPCR analysis and preamplification analysis. BL was supplier of samples. LL carried out the qPCR analysis and preamplification analysis. PP was responsible for verification of methylation data by HRM. RA assisted in verification of methylation data by HRM. SL was supplier of samples. PB revised the manuscript critically for important intellectual content. NA helped to draft the manuscript. PV, department chair, coordinated whole research and helped to draft the manuscript. All authors read and approved the final manuscript.

## Pre-publication history

The pre-publication history for this paper can be accessed here:

http://www.biomedcentral.com/1471-2350/15/17/prepub

## Supplementary Material

Additional file 1: Table S1a Primer sequences for each MMR gene for the amplification of M (methylated) and U (unmethylated) template DNA. **Table S1b:** Primers for MS-HRM. **Table S2.** mRNA expression levels of MMR genes in tumor and adjacent mucosal tissues in the study subjects. **Table S3a.** mRNA expression levels of MMR genes after stratification for tumor localization. **Table S3b.** mRNA expression levels of MMR genes in colon cancer. Click here for file

Additional file 2: Figure S1 *MLH1* promoter methylation presented in adjacent mucosal tissue in Patient 2 by MSP. (H = adjacent mucosal tissue, T = tumor tissue; U = amplified sequence with primers complementary to bisulfate converted unmethylated DNA sequence; M = amplified sequence with primers complementary to bisulfate converted methylated DNA seguence; NC = negative control; PC = positive control). Click here for file
