# Supplementary material for: Molecular characteristics of mismatch repair genes in sporadic colorectal tumors in Czech patients
Source: BMC Med Genet. 2014 Jan 31;15:17. doi: 10.1186/1471-2350-15-17 (PMC3913626; doi:10.1186/1471-2350-15-17)
Supplement: Additional file 1: Table S1a — Primer sequences for each MMR gene for the amplification of M (methylated) and U (unmethylated) template DNA. Table S1b: Primers for MS-HRM. Table S2. mRNA expression levels of MMR genes in tumor and adjacent mucosal tissues in the study subjects. Table S3a. mRNA expression levels of MMR genes after stratification for tumor localization. Table S3b. mRNA expression levels of MMR genes in colon cancer. [file 1471-2350-15-17-S1.doc]

**Additional file 1: Table S1a:** Primer sequences for each MMR gene for the amplification of M (methylated) and U (unmethylated) template DNA

| **Gene** | **Primer sequence** |
| --- | --- |
| *MLH1* | Forward M primer: TTT TTT TAG GAG TGA AGG AGG TTA C  Reverse M primer: ACT AAA CAC GAA TAC TAC GAA CGA T |
|  | Forward U primer: TTT TTT TAG GAG TGA AGG AGG TTA T  Reverse U primer: ACT AAA CAC AAA TAC TAC AAA CAA T |
| *MSH2* | Forward M primer: AGT AGT TTT TTT AGT GCG GAG GTA C  Reverse M primer: ACA AAT TCA AAT CCG AAA CGA |
|  | Forward U primer: GTA GTT TTT TTA GTG TGG AGG TAT GG  Reverse U primer: TAA TCA CAA ATT CAA ATC CAA AAC A |
| *MSH3* | Forward M primer: ATT CGA AAT ATT ATT TTT TGG GTT C  Reverse M primer: GAC TAA ATT CCC CTT TTC TAC TAC G |
|  | Forward U primer: TTG AAA TAT TAT TTT TTG GGT TTG A  Reverse U primer: CAA CTA AAT TCC CCT TTT CTA CTA CAC |
| *MLH3* | Forward M primer: TTT CGT GTT TTG GAT TTA GGT TC  Reverse M primer: CGA CAA CAA CTA ACG AAT AAA CGT |
|  | Forward U primer: TTT TGT GTT TTG GAT TTA GGT TTG  Reverse U primer: CCA ACA ACA ACT AAC AAA TAA ACA TT |
| *PMS1* | Forward M primer: TAG TAG GTT TGT CGC GTT GTT TAC  Reverse M primer: ACT AAA TTT TCT CTA ACC CAA AAC G |
|  | Forward U primer: AGT AGG TTT GTT GTG TTG TTT ATG A  Reverse U primer: TAA ATT TTC TCT AAC CCA AAA CAC T |
| *PMS2* | Forward M primer: TAG AAT TAA AGT AAA AGG GGG TAG C  Reverse M primer: CTA TCA AAA ATC GAC TTC GTA ACG T |
|  | Forward U primer: GGT AGA ATT AAA GTA AAA GGG GGT AGT  Reverse U primer: TAT CAA AAA TCA ACT TCA TAA CAT C |
| *MSH6* | Forward M primer: TTT TAG GAG TTT CGT TCG ATA GAA C  Reverse M primer: AAA CCT TAT TAA CAT CAC TCA ACG C |
|  | Forward U primer: TTT TAG GAG TTT TGT TTG ATA GAA TG  Reverse U primer: AAC CTT ATT AAC ATC ACT CAA CAC C |
| *EXO1* | Forward M primer: GCG GGT GTA AAT ATT TTT AAT TTT C  Reverse M primer: ATA AAC AAC GAC TCA CTA CCA TAC G |
|  | Forward U primer: TGG GTG TAA ATA TTT TTA ATT TTT GA  Reverse U primer: AAA CAA CAA CTC ACT ACC ATA CAA C |

**Additional file 1: Table S1b:** Primers for MS-HRM

| Gene | Primer sequences | Number of CpG-sites / length of amplified fragment [bp] | Ta  [°C] | Ref. |
| --- | --- | --- | --- | --- |
| *MLH1* | 5’-TCGAGTCGGGTTTATTTAAGGGTTA-3’ 5’- ACATACGCTATACATACCTCTACCC-3’ | 8 / 100 | 56 | Oster et al.,  Int J Cancer, 2011. |
| *MLH3* | 5'-GGATTTTAGGTGATTTTGGG-3'  5'-ACCATCATCAAAACCCTTCTA-3' | 4 / 85 | 56 | - |

bp, base pairs; Ta, annealing temperature

**Additional file 1: Table S2.** mRNA expression levels of MMR genes in tumor and adjacent mucosal tissues in the study subjects

| **Gene** |  | **Tissue (n=53)** |  | **Median** |  | **Percentiles (25-75)** |  | **P value** |  | **Fold change** |  |
| --- | --- | --- | --- | --- | --- | --- | --- | --- | --- | --- | --- |
|  |  |  |  |  |  |  |  |  |  |  |  |
| *EXO1* |  | Adjacent mucosa |  | 3.93 |  | 2.90-5.40 |  | **0.048** |  | 1.16 |  |
|  |  | Tumor |  | 4.86 |  | 3.86-6.11 |  |  |  |  |  |
|  |  |  |  |  |  |  |  |  |  |  |  |
| *MLH1* |  | Adjacent mucosa |  | 3.45 |  | 2.87-4.07 |  | 0.65 |  | 1.00 |  |
|  |  | Tumor |  | 3.41 |  | 2.83-4.27 |  |  |  |  |  |
|  |  |  |  |  |  |  |  |  |  |  |  |
| *MLH3* |  | Adjacent mucosa |  | 4.40 |  | 3.53-5.56 |  | 0.78 |  | -1.04 |  |
|  |  | Tumor |  | 4.29 |  | 3.70-5.50 |  |  |  |  |  |
|  |  |  |  |  |  |  |  |  |  |  |  |
| *MSH2* |  | Adjacent mucosa |  | 2.60 |  | 1.55-4.23 |  | 0.10 |  | 1.14 |  |
|  |  | Tumor |  | 3.57 |  | 2.32-4.62 |  |  |  |  |  |
|  |  |  |  |  |  |  |  |  |  |  |  |
| *MSH3* |  | Adjacent mucosa |  | 4.04 |  | 3.45-4.42 |  | 0.48 |  | 1.02 |  |
|  |  | Tumor |  | 4.12 |  | 3.60-4.44 |  |  |  |  |  |
|  |  |  |  |  |  |  |  |  |  |  |  |
| *MSH6* |  | Adjacent mucosa |  | 2.96 |  | 1.95-4.47 |  | 0.94 |  | 1.06 |  |
|  |  | Tumor |  | 3.36 |  | 2.55-4.37 |  |  |  |  |  |
|  |  |  |  |  |  |  |  |  |  |  |  |
| *PMS1* |  | Adjacent mucosa |  | 3.17 |  | 2.23-4.35 |  | 0.55 |  | 1.01 |  |
|  |  | Tumor |  | 3.63 |  | 2.64-4.54 |  |  |  |  |  |
|  |  |  |  |  |  |  |  |  |  |  |  |
| *PMS2* |  | Adjacent mucosa |  | 5.75 |  | 5.11-6.90 |  | 0.53 |  | 1.02 |  |
|  |  | Tumor |  | 6.23 |  | 5.36-7.08 |  |  |  |  |  |
|  |  |  |  |  |  |  |  |  |  |  |  |

*Fold change is calculated from mean values; Significant differences are in bold, differences significant after Dunn–Bonferroni correction (P<0.0032) are underlined.

**Additional file 1: Table S3a.** mRNA expression levels of MMR genes after stratification for tumor localization

|  |  |  |  | **Adjacent mucosal tissue** | | | | |  |  | **Tumor tissue** | | | | |  |
| --- | --- | --- | --- | --- | --- | --- | --- | --- | --- | --- | --- | --- | --- | --- | --- | --- |
| **Gene** |  | **Localization** |  | **Median** |  | **Percentiles (25-75)** |  | **P value** | **Fold change** |  | **Median** |  | **Percentiles (25-75)** |  | **P value** | **Fold change** |
|  |  |  |  |  |  |  |  |  |  |  |  |  |  |  |  |  |
| *EXO1* |  | Colon |  | 4.21 |  | 2.86-5.49 |  | 0.29 | 1.15 |  | 5.18 |  | 4.34-6.57 |  | **0.02** | 1.30 |
|  |  | Rectum |  | 3.53 |  | 2.90-4.94 |  |  |  |  | 4.41 |  | 3.04-5.15 |  |  |  |
|  |  |  |  |  |  |  |  |  |  |  |  |  |  |  |  |  |
| *MLH1* |  | Colon |  | 3.04 |  | 2.85-3.98 |  | 0.45 | -1.04 |  | 3.81 |  | 2.83-4.45 |  | 0.25 | 1.11 |
|  |  | Rectum |  | 3.69 |  | 3.08-4.07 |  |  |  |  | 3.35 |  | 2.81-4.08 |  |  |  |
|  |  |  |  |  |  |  |  |  |  |  |  |  |  |  |  |  |
| *MLH3* |  | Colon |  | 5.20 |  | 4.03-5.91 |  | 0.08 | 1.19 |  | 4.87 |  | 4.03-5.98 |  | 0.07 | 1.16 |
|  |  | Rectum |  | 4.10 |  | 3.47-5.12 |  |  |  |  | 4.13 |  | 3.25-5.07 |  |  |  |
|  |  |  |  |  |  |  |  |  |  |  |  |  |  |  |  |  |
| *MSH2* |  | Colon |  | 4.23 |  | 1.65-5.26 |  | **0.02** | 1.65 |  | 4.33 |  | 3.57-5.04 |  | **0.000** | 1.68 |
|  |  | Rectum |  | 2.23 |  | 1.48-3.20 |  |  |  |  | 2.66 |  | 1.87-3.72 |  |  |  |
|  |  |  |  |  |  |  |  |  |  |  |  |  |  |  |  |  |
| *MSH3* |  | Colon |  | 3.97 |  | 3.20-4.54 |  | 0.77 | -1.03 |  | 4.43 |  | 4.13-5.09 |  | **0.001** | 1.27 |
|  |  | Rectum |  | 4.05 |  | 3.49-4.42 |  |  |  |  | 3.76 |  | 3.29-4.19 |  |  |  |
|  |  |  |  |  |  |  |  |  |  |  |  |  |  |  |  |  |
| *MSH6* |  | Colon |  | 4.36 |  | 1.75-5.59 |  | 0.08 | -1.38 |  | 3.83 |  | 3.29-4.77 |  | **0.004** | 1.43 |
|  |  | Rectum |  | 2.45 |  | 1.98-3.79 |  |  |  |  | 2.74 |  | 2.14-4.06 |  |  |  |
|  |  |  |  |  |  |  |  |  |  |  |  |  |  |  |  |  |
| *PMS1* |  | Colon |  | 3.98 |  | 2.23-4.79 |  | 0.22 | 1.19 |  | 4.10 |  | 2.65-4.91 |  | 0.10 | 1.18 |
|  |  | Rectum |  | 2.90 |  | 2.23-4.10 |  |  |  |  | 3.27 |  | 2.57-4.05 |  |  |  |
|  |  |  |  |  |  |  |  |  |  |  |  |  |  |  |  |  |
| *PMS2* |  | Colon |  | 6.52 |  | 5.54-7.25 |  | **0.03** | 1.18 |  | 6.72 |  | 6.26-7.73 |  | **0.005** | 1.16 |
|  |  | Rectum |  | 5.35 |  | 4.86-6.61 |  |  |  |  | 5.72 |  | 5.11-6.36 |  |  |  |
|  |  |  |  |  |  |  |  |  |  |  |  |  |  |  |  |  |

* Fold change is counted from mean values; Significant differences are in bold, differences significant after Dunn–Bonferroni correction (P < 0.0032) are underlined.

**Additional file 1: Table S3b.** mRNA expression levels of MMR genes in colon cancer

|  |  | **Adjacent mucosal tissue** | |  | **Colon cancer** | |  |  |  |
| --- | --- | --- | --- | --- | --- | --- | --- | --- | --- |
| **Gene** |  | **Median** | **Percentiles (25-75)** |  | **Median** | **Percentiles (25-75)** |  | **P value** | **Fold change** |
|  |  |  |  |  |  |  |  |  |  |
| *EXO1* |  | 4.21 | 2.86-5.49 |  | 5.18 | 4.34-6.57 |  | 0.06 | 1.24 |
|  |  |  |  |  |  |  |  |  |  |
| *MLH1* |  | 3.04 | 2.85-3.98 |  | 3.81 | 2.83-4.45 |  | 0.30 | 1.08 |
|  |  |  |  |  |  |  |  |  |  |
| *MLH3* |  | 5.20 | 4.03-5.09 |  | 4.87 | 4.03-5.98 |  | 0.92 | -1.06 |
|  |  |  |  |  |  |  |  |  |  |
| *MSH2* |  | 4.23 | 1.65-5.26 |  | 4.33 | 3.57-5.03 |  | 0.34 | 1.15 |
|  |  |  |  |  |  |  |  |  |  |
| *MSH3* |  | 3.97 | 3.20-4.54 |  | 4.43 | 4.13-5.09 |  | **0.02** | 1.18 |
|  |  |  |  |  |  |  |  |  |  |
| *MSH6* |  | 4.36 | 1.75-5.59 |  | 3.83 | 3.29-4.77 |  | 0.72 | 1.08 |
|  |  |  |  |  |  |  |  |  |  |
| *PMS1* |  | 3.98 | 2.23-4.79 |  | 4.10 | 2.65-4.91 |  | 0.78 | 1.01 |
|  |  |  |  |  |  |  |  |  |  |
| *PMS2* |  | 6.52 | 5.54-7.25 |  | 4.72 | 6.26-7.43 |  | 0.42 | 1.01 |
|  |  |  |  |  |  |  |  |  |  |

* Fold change is counted from mean values; Significant differences are in bold, differences significant after Dunn–Bonferroni correction (P < 0.0032) are underlined.
