# Supplementary figures and images for: Molecular characteristics of mismatch repair genes in sporadic colorectal tumors in Czech patients
Source: BMC Med Genet. 2014 Jan 31;15:17. doi: 10.1186/1471-2350-15-17 (PMC3913626; doi:10.1186/1471-2350-15-17)

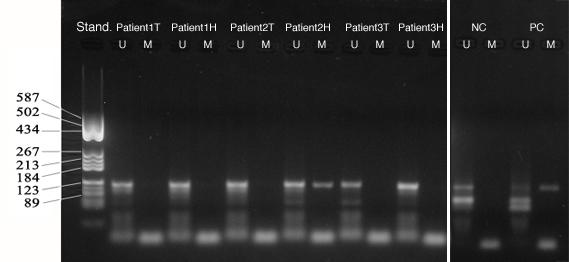

Supplement: Additional file 2: Figure S1 — MLH1 promoter methylation presented in adjacent mucosal tissue in Patient 2 by MSP. (H = adjacent mucosal tissue, T = tumor tissue; U = amplified sequence with primers complementary to bisulfate converted unmethylated DNA sequence; M = amplified sequence with primers complementary to bisulfate converted methylated DNA seguence; NC = negative control; PC = positive control). [file 1471-2350-15-17-S2.png]
